# Asymptomatic colo-ovarian fistula amidst acute psychosis: a case report

**DOI:** 10.1093/jscr/rjad525

**Published:** 2023-10-25

**Authors:** Meghan R Mansour, Steven A Kessler, Ali Khreisat, Justin K Skrzynski

**Affiliations:** Department of Medical Education, Oakland University William Beaumont School of Medicine, Rochester Hills, MI 48309, United States; Department of Medical Education, Oakland University William Beaumont School of Medicine, Rochester Hills, MI 48309, United States; Department of Internal Medicine, Corewell Health William Beaumont University Hospital, Royal Oak, MI 48703, United States; Department of Internal Medicine, Corewell Health William Beaumont University Hospital, Royal Oak, MI 48703, United States

**Keywords:** colo-ovarian fistula, abscess, psychosis, diverticular disease, diverticulitis, surgery, gynecologic surgery, colorectal surgery

## Abstract

This paper presents a rare case of an asymptomatic colo-ovarian fistula in a 45-year-old female with acute psychosis and a history of bipolar disorder, seizure disorder and substance misuse. The intricate diagnostic challenges arising from the patient’s complex medical history underscore the significance of a multidisciplinary approach. The absence of typical gastrointestinal symptoms and the presence of a tubo-ovarian abscess complicated the diagnosis of acute on chronic sigmoid diverticulitis and colo-ovarian fistula. Surgical intervention, including sigmoid resection, anastomosis and left salpingo-oophorectomy, led to successful resolution. This case highlights the need for further understanding of colo-ovarian fistula pathophysiology, improved diagnostic strategies, and the nuanced interplay between medical and psychiatric conditions in complex clinical scenarios.

## Introduction

Fistula formation is a recognized complication in approximately 10–13% of cases of diverticular disease [1]. Inflammatory responses to diverticular perforation or rupture may result in adhesions and fistula formation. A majority of reported cases describe symptoms that mirror those of the underlying disorder before specific signs of a fistula emerge; however, asymptomatic disease has also been less commonly documented [1, 2].

Colo-ovarian fistulas have rarely been described in current literature. Documented cases are most frequently reported in the context of primary ovarian neoplasms, ovarian abscesses, Crohn’s disease, and less often colonic diverticulitis [3, 4]. We present a case of an asymptomatic colo-ovarian fistula in the setting of a tubo-ovarian abscess and acute on chronic diverticulitis in a patient with acute psychosis.

## Case report

A 45-year-old female with a significant past medical history of bipolar disorder, seizure disorder and substance misuse was referred to the hospital for altered mental status. She reported a questionable history of metastatic cervical and pancreatic malignancies that could not be confirmed by outside records. She also recounted 8 years of cancer treatment that appeared to be absent from her medical records. Upon arrival, vital signs were stable and labs were unremarkable. Haloperidol was administered to manage her agitation, and a diagnosis of acute psychosis linked to bipolar disorder was established.

**Figure 1:**
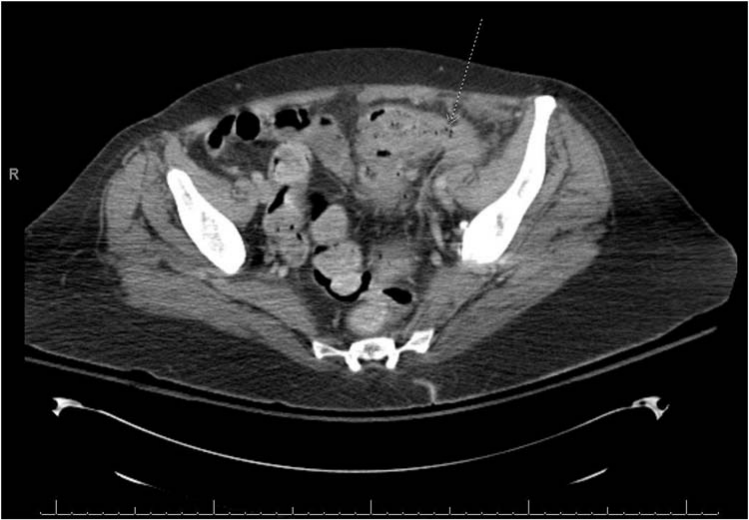
A fistulous connection from the sigmoid colon to the ovary is suggested. A dotted line arrow points out the connection.

**Figure 2:**
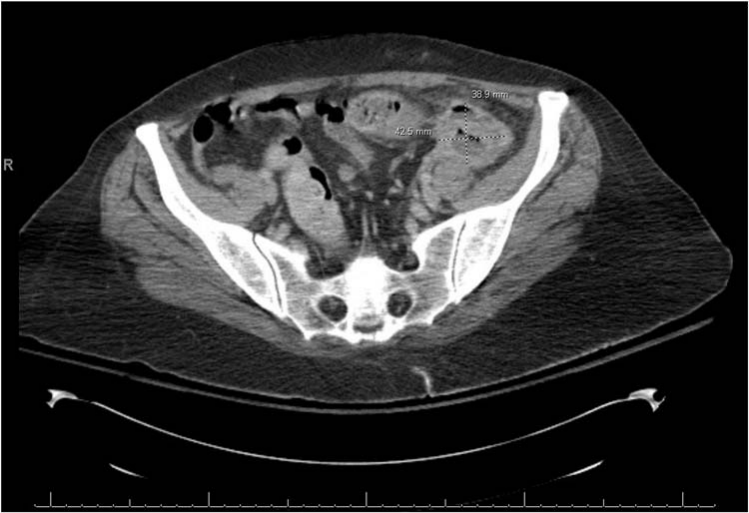
There is a thick-walled enhancing, 3.9 × 4.3 × 4.2 cm^3^ perisigmoidal collection containing nondependent gas and fluid with adjacent inflammatory fat stranding, suggestive of tubo-ovarian abscess.

There was a suspicion of underlying abdominal infection causing her altered mental status given the presence of abdominal pain and low grade fever. A computed tomography (CT) scan of the chest, abdomen and pelvis was completed to assess for prior or current malignancy. Acute on chronic sigmoid diverticulitis with a left colo-ovarian fistula and a 4.3 cm left adnexal abscess was found (Figs 1 and 2); yet, no evidence of malignancy was noted. Antibiotic therapy was initiated, and interventional radiology was consulted for abscess drainage. Cancer antigen (CA)-125 and carcinoembryonic antigen (CEA) were only mildly elevated and attributed to generalized inflammation.

She underwent CT-guided drainage of the abscess, extracting 10 cc of purulent fluid and insertion of a drainage catheter. Following the procedure, her agitation subsided, and she experienced only mild abdominal discomfort. She was discharged to a subacute rehabilitation facility 14 days after admission, with normal mental status and an abdominal drain in place.

She was readmitted for acute psychosis, 8 days following discharge. A follow-up CT scan of the abdomen and pelvis revealed progressive reduction in inflammatory changes and complete resolution of the abscess. The drain was removed at this time. Subsequently, twenty-three days after initial discharge, the patient was readmitted for the second time regarding worsening abdominal pain. Outpatient CT scan revealed redemonstration of the abscess and acute diverticulitis. She was again treated with intravenous antibiotics, without any relief in symptoms.

The patient underwent an uncomplicated exploratory laparotomy involving sigmoid resection with anastomosis and left salpingo-oophorectomy. The post-operative recovery was complicated by respiratory failure requiring ventilator support due to mucus plugging and atelectasis. However, the patient’s condition improved, and she was discharged home in a stable condition one week following the surgery.

## Discussion

This complicated clinical scenario of a 45-year-old female initially presenting with altered mental status highlights the challenges in diagnosing and managing patients with intricate medical histories and the importance of a multidisciplinary approach in such cases. The patient’s history of bipolar disorder, seizure disorder, and substance misuse adds a layer of complexity to her presentation. It is well established that individuals with bipolar disorder are at an increased risk of various medical comorbidities, which can complicate the diagnostic process [5].

The absence of classic abdominal tenderness or gastrointestinal symptoms further confounds the clinical picture, emphasizing the variability in presentation and the need for a high index of suspicion. The absence of gastrointestinal symptoms might have been influenced by the patient’s altered mental status or masked by her psychiatric history. The patient’s history of questionable malignancies was the only indication to order a CT scan, yet the subsequent discovery of acute on chronic sigmoid diverticulitis with a left colo-ovarian fistula and an adnexal abscess adds to the diagnostic challenge.

Diverticular disease is particularly prevalent among the elderly, especially individuals aged 60 and above [2, 6]. Notably, the patient in our case is younger than the classically observed patient population. Chronic diverticulitis, characterized by inflammation of colonic diverticula, likely created a localized environment conducive to the formation of a fistulous tract. Sigmoid diverticulitis, while common, rarely presents with colo-ovarian fistulas and abscesses. The proximity of the colon and ovaries in the pelvic cavity, exacerbated by the presence of a tubo-ovarian abscess, could have facilitated the abnormal communication between these structures. It is difficult to determine the precise aetiology of her colo-ovarian fistula, especially given the lack of an ovarian mass and the absence of gastrointestinal or diverticulitis symptoms. Previous cases have not described one forementioned aetiology as more common than the other [2–4, 7, 8].

The rarity of this condition hinders the establishment of a standardized imaging algorithm for suspected colo-ovarian fistulas [9]. Although no diagnostic imaging method has been deemed elite, a comprehensive analysis suggests that a range of diagnostic approaches, including pelvic ultrasound, CT scan, colonoscopy and barium enema, have all demonstrated successful utilization in diagnosis and perioperative planning [4, 8, 9]. Operative management options vary, with resection and primary anastomosis being the preferred choice. Other options include resection with primary anastomosis followed by prophylactic diversions, the Hartmann procedure and three-stage procedures [8, 10]. In our case, CT-drainage of the abscess resulted in recurrence, and a sigmoid resection with anastomosis and left salpingo-oophorectomy resulted in a final resolution, as supported by the literature.

In conclusion, the pathophysiology underlying the development of an asymptomatic colo-ovarian fistula in this case remains intriguing. This case underscores the need for further research to elucidate the precise mechanisms driving the formation of such fistulas, as well as to determine the best diagnostic approach and treatment options. It also highlights the intricate interplay between medical and psychiatric conditions, diagnostic challenges and the importance of a multidisciplinary approach.

## Data Availability

The data that support the findings of this study are available in this manuscript.
